# The influence of the Beijing Winter Olympic games on the demand for winter sports: An empirical analysis based on the Baidu Index

**DOI:** 10.1016/j.heliyon.2023.e20426

**Published:** 2023-09-26

**Authors:** Peipei Wu, Xiaochuan Zhu, Shuqian Yang, Junpei Huang

**Affiliations:** a College of Business Administration, Shanghai Business School, Shanghai, 201400, China; b School of Tourism, Shanghai Normal University, Shanghai, 200233, China; c School of Environmental and Geographical Sciences, Shanghai Normal University, Shanghai, 200233, China; d Department of Physical Education, Tongji University, Shanghai, 200092, China

**Keywords:** Baidu index, Beijing winter olympics, Major sports events, Spatial-temporal characteristics, Winter sports

## Abstract

**Background:**

The 2022 Beijing Winter Olympics is a representative large-scale sporting event, which not only promotes the development of the national and regional economy, society, and culture but also increases the demand of residents for winter sports, thus helping achieve the grand goal of “encouraging 300 million people to participate in winter sports.” This research explores the influence of the Beijing Winter Olympics on residents’ demand for winter sports in the Beijing–Tianjin–Hebei and Yangtze River Delta urban agglomerations in China.

**Methods:**

Applying big data mining techniques, the Baidu Index of Winter Olympics-related terms are used to measure residents' interest in the Beijing Winter Olympics, and the ratio of the Baidu Index of five winter sports (ice skating, ice hockey, curling, luge, and skiing) to the number of internet searches is used to capture residents’ demand for winter sports. Moreover, we explore the spatial–temporal pattern of the interest in the Winter Olympics and the demand for winter sports and construct an econometric model to test the driving effect of the Winter Olympics empirically.

**Results:**

The results show that 1) since 2011, interest in Winter Olympics has been on the rise, and the interest of residents in Beijing–Tianjin–Hebei has been higher than that of the Yangtze River Delta; 2) the demand for skating and skiing, which are two popular winter sports, shows a declining geographical concentration, indicating that the popularity of these two sports is on the increase; 3) the demand for winter sports in the peripheral cities in Beijing–Tianjin–Hebei shows a trend of specialization, while Beijing, Tianjin, and some cities in the Yangtze River Delta present a trend of diversification; and 4) the interest in the Beijing Winter Olympics influences the demand for winter sports positively.

**Conclusion:**

This study shows that the increase in interest in the Beijing Winter Olympics boosts residents’ demand for winter sports, which implies that hosting Winter Olympics successfully drives winter sports participation in China.

## 1 Introduction

The National Fitness Program implemented in China is not only the top priority in promoting the development of the sports industry, but also the key link to improve people's quality of life, which is an indispensable part of the strategy of building China into a leading sporting nation. The bid to host the Beijing Winter Olympic Games also provides an excellent opportunity to improve the participation of the residents in winter sports. Since the successful application for the 2015 Beijing Winter Olympics, winter sports and ice and snow culture have received wide interest in China. Getting more people involved in winter sports and promoting the construction of a leading sporting nation and healthy China is the starting point to host the Beijing Winter Olympics. The *Development Plan of Winter Sports (2016–2025)* proposes to drive 300 million people to participate in winter sports by 2025, and the *Opinions on Taking the 2022 Beijing Winter Olympics as an Opportunity to Vigorously Develop Winter Sports* suggests enhancing the profile and popularity of winter sports among residents.

In recent years, China has made considerable achievements in the promotion of winter sports; however, there are still some problems such as low mass participation, uneven participation among regions, and uneven development of different sports which slow down the promotion of the National Fitness Program and the popularization of winter sports. Therefore, in the context of the successful hosting of the 2022 Beijing Winter Olympic Games, it is necessary to clarify the influence of major events on the demand for winter sports, and how to meet and improve it.

The influence of sporting events has been studied extensively from the perspectives of the country, city, community, and residents. At the national level, scholars have highlighted that the hosting of sporting events can not only enhance the country's popularity abroad [1] but also promote the national identity of domestic residents [2]. At the city level, it plays a role in raising the profile of the city [3], as well as promoting urban economic development [4,5], boosting urban renewal [6] and enhancing the urban resilience [7], specifically the promotion of urban tourism [[8], [9], [10]]. At the level of community and residents, holding sporting events improves residents' sense of acquisition [11], subjective well-being [12] and quality of life [13], and enhances their support for events [14] and prosocial behavior [15], thus forming a collective attachment [16,17] and local attachment [18].

As an important means of promoting sports to benefit the people and improving the national sense of gain and happiness in our country, increasing numbers of studies have focused on discussing the influence of sporting events on residents’ sports participation in recent years. They concluded that by participating in sports activities, residents can keep fit [19], improve their academic performance [20], and improve their well-being [21]. In the current era of ultrafast urbanization and rapid flow of human resources between regions, scholars have also reported that sports participation can improve psychological and social inclusion [22]. As for individual characteristics, scholars have analyzed the influence of parents and family culture [23,24], age [25], gender [26,27], marital status [28], educational background [29], occupation and income [30], motivation [31], habits [32], and satisfaction with previous participation in physical activities [33]. Furthermore, in terms of regional characteristics, some studies focused on the effect of factors such as sports facilities [34], and neighborhood environment [35]. Winter sports in particular are affected by climate [36,37].

Generally, the research on the effect of sporting events and the influencing factors of sports participation are academic hotspots. However, most of the research on the influence of sporting events is at the national and city levels, while the research at the individual level mostly focuses on the exploration of residents' feelings, and the research on the influence of sports participation is still somewhat insufficient. Additionally, related research mostly uses the data obtained from questionnaires and social surveys [[38], [39], [40]], but seldom uses new data such as web texts and internet indexes. However, with the rapid development of the internet and technologies including big data and artificial intelligence, internet search data have been used increasingly widely in disciplines such as economics and management, relying on its powerful real-time. The few studies on sports and winter sports using internet indexes mainly focus on the discussion of the description of the index [41], lacking interpretation and extension of the meaning underlying the data as well as the exploration of the influencing factors. At present, China is in a critical period to stimulate the participation of the public in winter sports; thus, the issues the influence of major sporting events on residents’ participation in winter sports, the difference in the influence on different kinds of winter sports, the regional heterogeneity factors between different cities, and how to promote and release the demand for winter sports are worthy of further discussion.

To address these questions, in this study, we empirically explore the influence of the Beijing Winter Olympics on residents' demand for winter sports in the Beijing–Tianjin–Hebei and Yangtze River Delta urban agglomerations in China. Applying big data mining techniques, the Baidu Index of Winter Olympics-related terms is used to measure residents' interest in the Beijing Winter Olympics, and the ratio of the Baidu Index of five winter sports (ice skating, ice hockey, curling, luge, and skiing) to the number of internet searches is used to capture residents' demand for winter sports. Furthermore, we explore the spatial–temporal pattern of the interest in the Winter Olympics and the demand for winter sports and construct an econometric model to test the driving effect of the Winter Olympics empirically. The results show that the increased interest in the Winter Olympics boosts residents’ demand for winter sports significantly, implying that the success of the Winter Olympics drives participation in winter sports.

## 2 Methodology

### 2.1 Study area

**Fig. 1:**
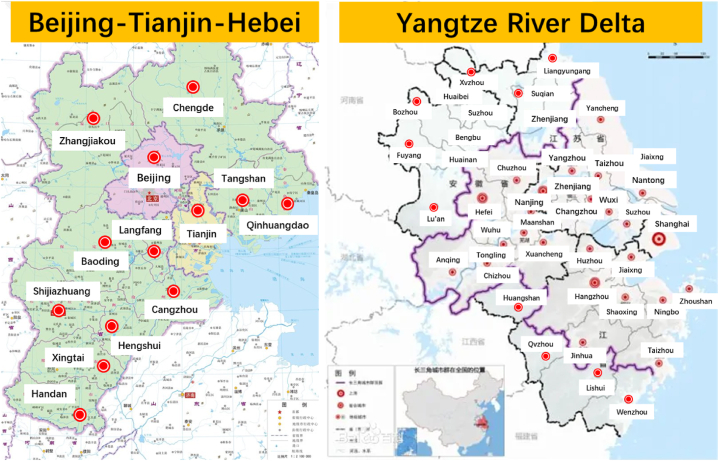
Study area.

The study area in this paper comprised the Beijing–Tianjin–Hebei and Yangtze River Delta urban agglomerations, both of which are state-level urban agglomerations. The Beijing–Tianjin–Hebei urban agglomeration is located in the north of China, and the climatic conditions there are conducive to the development of winter sports. Furthermore, Beijing, as the host of the Winter Olympics, has a remarkable effect of driving residents in urban agglomerations to watch and participate in winter sports. The Yangtze River Delta is characterized by active economic development and a high degree of openness to the outside world. Moreover, people show great interest in the popular events represented by winter sports, and the data changes are more representative. According to the *Outline of Coordinated Development of the Beijing*–*Tianjin*–*Hebei Region* and *Outline of the Integrated Regional Development of the Yangtze River Delta*, the study area of this paper is presented in Table 1 and Fig. 1.

**Table 1:** Cities in Beijing-Tianjin-Hebei region and Yangtze River Delta.

urban agglomeration	city	number
Beijing–Tianjin–Hebei	Beijing Tianjin Hebei Province: Baoding, Tangshan, Shijiazhuang, Langfang, Qinhuangdao, Zhangjiakou, Chengde, Cangzhou, Hengshui, Xingtai, Handan	13
Yangtze River Delta	Shanghai Jiangsu Province: Nanjing, Wuxi, Xuzhou, Changzhou, Suzhou, Nantong, Lianyungang, Huai'an, Yancheng, Yangzhou, Zhenjiang, Taizhou, Suqian Zhejiang Province: Hangzhou, Ningbo, Wenzhou, Jiaxing, Huzhou, Shaoxing, Jinhua, Quzhou, Zhoushan, Taizhou, Lishui Anhui Province: Hefei, Wuhu, Bengbu, Huainan, Maanshan, Huaibei, Tongling, Anqing, Huangshan, Chuzhou, Fuyang, Suzhou, Lu'an, Bozhou, Chizhou, Xuancheng	41

### 2.2 Data

The research time ranges from January 1, 2011, to March 31, 2022, which covers all the stage of bidding, preparation, and hosting stages of the Beijing Winter Olympic Games.

Data on residents' demand for winter sports and the interest shown in the Beijing Winter Olympics comes from the Baidu Index (https://index.baidu.com). The Baidu Index is a data analysis platform based on the massive amount of internet users' behavior data of Baidu (the world's largest Chinese search engine, https://www.baidu.com/), which is one of the most important statistical analysis platforms on the internet in mainland China. Since its release, the Baidu Index has become an important basis for many enterprises' marketing decisions and is widely used in academic research in fields including economics [[42], [43], [44]], tourism [45,46], and public health [47,48]. The Baidu search index is based on the search volume of internet users on the Baidu website, with keywords as the statistical object, and scientifically analyzing and calculating the weighted sum of the search frequency of each keyword in the Baidu website.

Data on air quality, average temperature, and temperature difference come from Weather Post Report (http://www.tianqihoubao.com). Data on the number of fixed broadband users, and GDP per capita comes from the *China City Statistical Yearbook* (2011–2020) and the statistical yearbooks and bulletins of each city in the study area.

### 2.3 Measurement of the spatial characteristics of the demand for winter sports

In this study, the ratio of the Baidu Index of five winter sports, ice skating, ice hockey, curling, luge, and skiing to the number of internet searches is selected as residents' demand for winter sports, and the sum of five subdivided demand is considered as the total demand for winter sports. The reason why the Baidu Index of winter sports is chosen as the measurement of the demand for winter sports is that it reflects users' search volume for winter sports, and demand is the basis of the search. There is a positive correlation between search and demand; thus, the Baidu Index can be used as a proxy variable for residents’ demand.

Referring to the methods of geographic concentration of industries and regional industrial agglomeration, this study explores the spatial characteristics of residents’ demand for winter sports in two dimensions: geographic concentration of demand and urban specialization of demand. Drawing on different applications of the Hirschman–Herfindahl Index to industrial geographic concentration and regional industrial agglomeration, the formula for calculating the geographical concentration index of the demand for winter sports is constructed as equation (1):

(1) $$Hh_{j,t}=\sum_{i=1}^{n}{\left(\frac{b_{i,j,t}}{b_{j,t}}\right)}^{2}$$

Here, *i* denotes city, and *j* denotes certain winter sports including ice skating, ice hockey, curling, luge, and skiing. Furthermore, $Hh_{j,t}$ denotes the geographical concentration index of the demand for winter sports *j* in year *t*, $b_{i,j,t}$ denotes the demand for winter sports *j* in city *i* in year *t*, and $b_{j\cdot t}$ denotes the total demand for winter sports *j* of all cities in year *t*, and The closer $Hh_{j,t}$ is to 1, the more concentrated the geographical distribution of the demand for winter sports *j* in the region. This indicates a weaker geographical concentration of the demand for winter sports j in the region.

Then, the formula for calculating the urban specialization index of demand for winter sports is as equation (2):

(2) $$Hh_{i,t}=\sum_{j=1}^{m}{\left(\frac{b_{i,j,t}}{b_{i,t}}\right)}^{2}$$

Here, $Hh_{i\cdot t}$ denotes the specialization index of the demand for winter sports in city *i* in year t, and $b_{i\cdot t}$ denotes the total demand for winter sports in city i in year *t*. When $Hh_{i,t}$ is close to 1, it means that city i is more concentrated on certain kinds of winter sports, that is, the degree of specialization of winter sports is high. Otherwise, it indicates that the demand for winter sports in city *i* is relatively average, implying that the degree of specialization of winter sports is low and shows high diversification.

### 2.4 Empirical model

To further elucidate the influence of the Beijing Winter Olympics on the demand for winter sports, an econometric model is constructed as follows:

(3) $${PIS}_{k,i,t}=\alpha +{\beta OWG}_{i,t}+\gamma_{1}{GDP}_{i,t}+\gamma_{2}{NET}_{i,t}+\gamma_{3}{ARK}_{i,t}+\gamma_{4}{TEA}_{i,t}+\gamma_{5}{TED}_{i,t}+\varepsilon_{i,t}$$

Here, ${PIS}_{i,t}$ is the explained variable of this study, which indicates the residents' demand for winter sports *k* in city *i* in year *t*. The ratio of the Baidu Index of five winter sports, ice skating, ice hockey, curling, luge, and skiing, to the number of internet searches is selected as the proxy of residents' demand for winter sports, and the sum of five subdivided demand is taken as the total demand for winter sports. ${OWG}_{i,t}$ is the core explanatory variable of this study, which indicates the interest in the Beijing Winter Olympics in city *i* in year *t*. It is measured by the average of the Baidu Index for “Beijing Winter Olympics” and “Winter Olympics” divided by the number of internet users. β is the coefficient of interest, if β>0, this indicates that the increased interest in the Winter Olympics improves residents' demand for winter sports, i.e., the holding of the Winter Olympics can drive residents’ participation in winter sports and vice versa.

In addition to large-scale sporting events, residents’ individual and regional characteristics also affect participation in sports activities. Therefore, this study selects regional economic development (${GDP}_{i,t}$), the proportion of internet mobile terminals (${NET}_{i,t}$), and climatic conditions (${ARK}_{i,t}$, ${TEA}_{i,t}$, ${TED}_{i,t}$) as control variables. According to the uniqueness of winter sports, we further divide climatic conditions into air quality (${ARK}_{i,t}$), average temperature (${TEA}_{i,t}$), and temperature difference (${TED}_{i,t}$). $\gamma_{1}-\gamma_{5}$ is the estimated coefficient of the control variables.

The greater the regional economic development, the stronger the residents' consumption power and leisure demands; thus, the regional economy has a positive effect on the demand for winter sports. The proportion of mobile internet reflects residents’ need for entertainment on mobile internet, and the higher the mobile internet index, the greater the proportion of mobile users, implying residents will spend more time on leisure activities that rely on mobile internet, such as short videos and mobile games, besides searching for winter sports, which are substitutes for winter sports. Thus, it has a negative impact on the demand for winter sports. Winter sports have certain requirements in terms of climatic conditions, in which air quality positively influences the demand for winter sports, while average air temperature and temperature difference have a negative impact on it. Therefore, it is expected that the coefficient of $\beta ,\gamma_{1},\gamma_{3}$ is positive and $\gamma_{2}、\gamma_{4}、\gamma_{5}$ is negative.

The economic development is measured by GDP per capita, and the proportion of internet mobile terminals is captured by the mobile terminal Baidu Index of winter sports divided by the whole Baidu Index of winter sports (including mobile terminals and computer terminals). We selected the log of average PM2.5 as the proxy of air quality, the annual average temperature as the proxy of average temperature, and the standard deviation of the temperature within one year as the proxy of the temperature difference.

## 3 Spatial–temporal characteristics of interest in the Beijing Winter Olympics

**Fig. 2:**
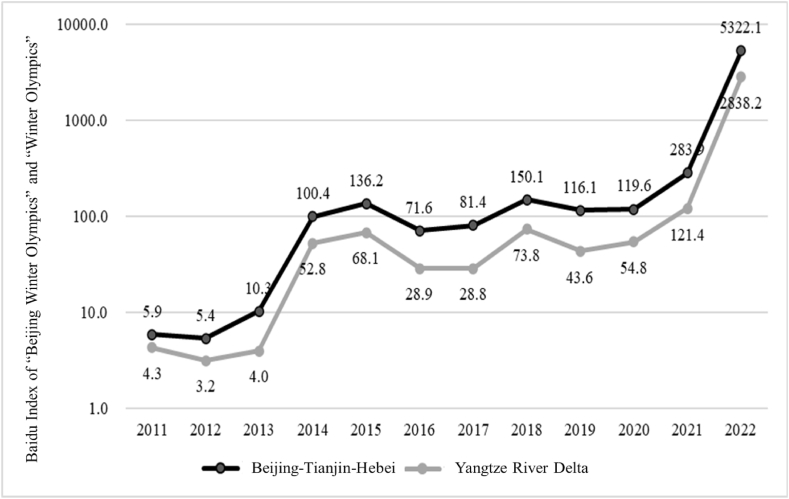
The annual trend of the attention to 2022 winter olympics from 2011 to 2022.

This study selected the Baidu Index of “Beijing Winter Olympics” and “Winter Olympics” in 51 cities in the two urban agglomerations from 2011 to 2022 and calculated the average value of each city over the years according to the major urban agglomerations, as shown in Fig. 2.

Generally, the residents of the two urban agglomerations showed little interest in Winter Olympics-related keywords before 2014. Since China proposed to bid for the Beijing Winter Olympics in 2014, the residents' interest in the Winter Olympics in China gradually increased and reached a small peak in 2015 when the Olympic bid was successful, and then began to fall back. By the time of the 2018 PyeongChang Winter Olympics, Chinese residents' interest in the Winter Olympics had been further enhanced. With the introduction and construction of policies in recent years, residents’ interest in the Winter Olympics and the Beijing Winter Olympics has been relatively stable, and until the successful hosting of the Beijing Winter Olympics in 2022, the interest in the Winter Olympics had reached its peak. From a comparison of the Beijing–Tianjin–Hebei and the Yangtze River Delta, the residents in Beijing–Tianjin–Hebei were more interested in the Winter Olympics than those in the Yangtze River Delta, and the growth trend remains the same. Before the bid for the Beijing Winter Olympics, the difference between them was small, while with the bid, preparation, and hosting of the Beijing Winter Olympics, the gap between them widened further.

**Fig. 3:**
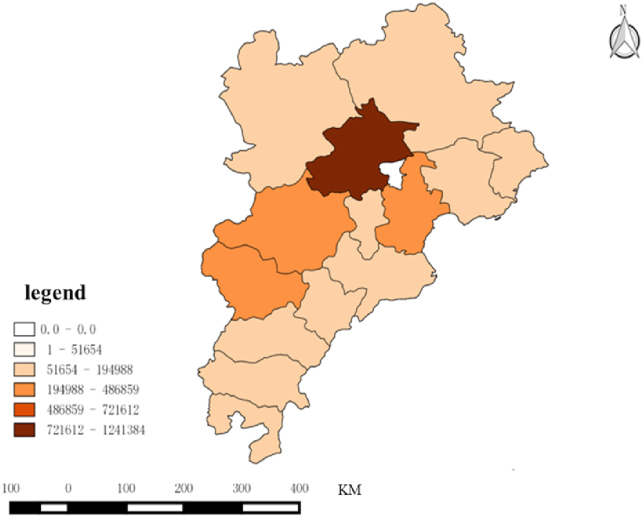
Spatial characteristics of the attention to Beijing Winter olympics in Beijing–Tianjin–Hebei region.

**Fig. 4:**
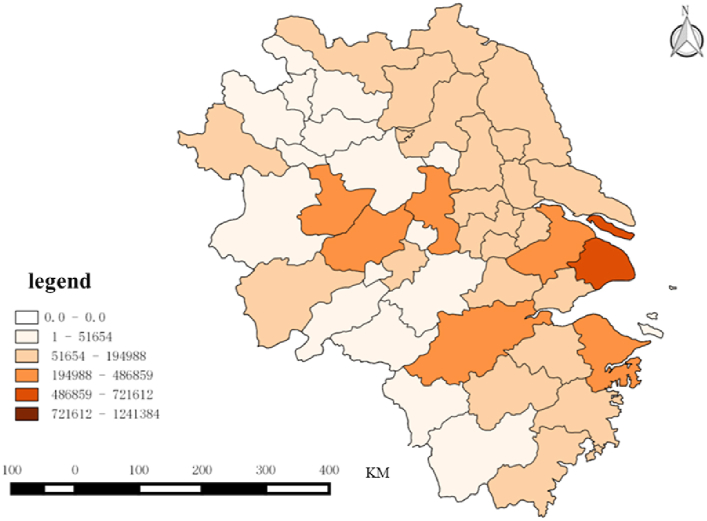
Spatial characteristics of the attention to Beijing Winter olympics in Yangtze River Delta region.

In terms of spatial characteristics, by comparing the spatial distribution of the Beijing–Tianjin–Hebei and the Yangtze River Delta, it can be observed that the cities with the greatest interest in the two urban agglomerations were the core cities, namely, Beijing and Shanghai, as illustrated in Fig. 3, Fig. 4. As the host of the 2022 Winter Olympics, Beijing was greatly influenced by it, and residents took great interest in the Winter Olympics. As the core city in the Yangtze River Delta, Shanghai has played a leading role in the surrounding areas for a long time, and as the economic center of the country, its residents’ consumption level is high, and they show more interest in sporting events at home and abroad.

## 4 Spatial–temporal characteristics of the demand for winter sports

### 4.1 Overall time trend

In this study, the ratio of the Baidu Index of five winter sports, ice skating, ice hockey, curling, luge, and skiing, to the number of internet access is selected as residents’ demand for winter sports, the trend of the demand for winter sports in the two urban agglomerations is as shown in Table 2.

**Table 2:** The trend of demand for winter sports from 2011 to 2022.

sports	skating					
region	nationwide		Beijing-Tianjin-Hebei Region		Yangtze River Delta Region	
year	Baidu Index	growth rate	Baidu Index	growth rate	Baidu Index	growth rate
2011	11.50	–	16.94	–	9.69	–
2012	10.87	−5.60%	17.95	5.78%	8.51	−12.92%
2013	14.13	26.08%	22.98	24.59%	11.18	27.11%
2014	20.77	38.03%	34.03	38.75%	16.35	37.57%
2015	20.32	−2.19%	33.32	−2.09%	15.99	−2.26%
2016	21.46	5.47%	33.38	0.15%	17.49	8.98%
2017	24.57	13.49%	38.85	15.16%	19.81	12.45%
2018	28.20	13.76%	44.00	12.44%	22.93	14.61%
2019	27.74	−1.63%	44.82	1.83%	22.05	−3.93%
2020	32.63	16.19%	53.86	18.33%	25.55	14.73%
2021	34.43	5.39%	53.69	−0.31%	28.01	9.17%
2022	71.60	70.11%	94.35	54.93%	64.02	78.25%
mean	26.52	16.28%	40.68	15.42%	21.80	16.70%
sports	ice hockey					
region	nationwide		Beijing-Tianjin-Hebei Region		Yangtze River Delta Region	
year	Baidu Index	growth rate	Baidu Index	growth rate	Baidu Index	growth rate
2011	10.40	–	14.69	–	8.97	–
2012	10.99	5.55%	15.87	7.75%	9.36	4.28%
2013	15.54	34.29%	21.44	29.82%	13.57	36.71%
2014	26.64	52.65%	38.78	57.60%	22.60	49.93%
2015	23.29	−13.43%	34.09	−12.87%	19.69	−13.76%
2016	23.01	−1.20%	33.16	−2.78%	19.62	−0.33%
2017	31.25	30.38%	45.38	31.12%	26.55	30.00%
2018	34.23	9.09%	49.63	8.95%	29.10	9.17%
2019	34.89	1.93%	53.88	8.22%	28.57	−1.86%
2020	40.29	14.36%	60.26	11.18%	33.64	16.32%
2021	43.07	6.66%	64.99	7.54%	35.76	6.10%
2022	135.12	103.32%	190.02	98.06%	116.82	106.26%
mean	35.73	22.14%	51.85	22.24%	30.35	22.07%
sports	curling					
region	nationwide		Beijing-Tianjin-Hebei Region		Yangtze River Delta Region	
year	Baidu Index	growth rate	Baidu Index	growth rate	Baidu Index	growth rate
2011	23.06	–	26.69	–	21.86	–
2012	19.57	−16.37%	25.87	−3.12%	17.47	−22.31%
2013	28.63	37.58%	37.11	35.71%	25.80	38.49%
2014	55.72	64.23%	77.08	70.01%	48.60	61.31%
2015	40.22	−32.32%	56.84	−30.23%	34.68	−33.44%
2016	37.30	−7.53%	53.46	−6.12%	31.90	−8.33%
2017	44.63	17.90%	64.09	18.09%	38.15	17.84%
2018	48.91	9.15%	70.05	8.88%	41.87	9.30%
2019	40.47	−18.89%	67.24	−4.09%	31.54	−28.14%
2020	45.46	11.62%	76.84	13.33%	35.01	10.42%
2021	58.81	25.60%	94.54	20.66%	46.89	29.00%
2022	246.00	122.83%	371.87	118.92%	204.03	125.26%
mean	57.40	19.44%	85.14	22.00%	48.15	18.13%
sports	luge					
region	nationwide		Beijing-Tianjin-Hebei Region		Yangtze River Delta Region	
year	Baidu Index	growth rate	Baidu Index	growth rate	Baidu Index	growth rate
2011	11.36	–	14.17	–	10.42	–
2012	11.65	2.52%	15.54	9.20%	10.35	−0.71%
2013	13.43	14.20%	16.83	7.99%	12.29	17.16%
2014	18.31	30.77%	23.97	34.98%	16.42	28.81%
2015	16.96	−7.65%	23.01	−4.06%	14.94	−9.44%
2016	16.56	−2.40%	21.94	−4.76%	14.76	−1.23%
2017	18.99	13.68%	25.42	14.70%	16.85	13.22%
2018	22.45	16.72%	28.67	12.01%	20.39	18.99%
2019	24.08	6.98%	31.32	8.84%	21.66	6.07%
2020	25.08	4.09%	34.07	8.41%	22.09	1.97%
2021	26.80	6.60%	38.51	12.25%	22.89	3.52%
2022	75.47	95.19%	106.05	93.43%	65.27	96.16%
mean	23.43	16.43%	31.62	17.54%	20.69	15.86%
sports	skiing					
region	nationwide		Beijing-Tianjin-Hebei Region		Yangtze River Delta Region	
year	Baidu Index	growth rate	Baidu Index	growth rate	Baidu Index	growth rate
2011	20.34	–	36.97	–	14.81	–
2012	21.74	6.61%	39.78	7.33%	15.71	5.95%
2013	35.85	49.02%	55.23	32.52%	29.38	60.61%
2014	52.67	38.01%	89.07	46.90%	40.55	31.94%
2015	59.46	12.11%	94.99	6.43%	47.62	16.06%
2016	46.59	−24.28%	76.60	−21.43%	36.58	−26.23%
2017	46.28	−0.66%	76.96	0.47%	36.07	−1.41%
2018	58.30	22.98%	85.97	11.05%	49.09	30.58%
2019	55.02	−5.79%	82.68	−3.90%	45.80	−6.93%
2020	47.79	−14.06%	74.71	−10.13%	38.83	−16.47%
2021	53.99	12.17%	81.60	8.82%	44.78	14.23%
2022	108.90	67.42%	150.26	59.23%	95.11	71.96%
mean	50.58	14.87%	78.74	12.48%	41.19	16.39%

Overall, from 2011 to 2022, the residents' interest in the five winter sports showed a fluctuating upward trend, similar to the residents’ interest in the Winter Olympics. In 2014 and 2022, when the Winter Olympics were bid for and held, interest in winter sports showed great growth. Comparing the five sports, curling and skiing were found to have relatively high demand as sports attracting high interest among residents, followed by ice hockey, while skating and luge had relatively low public awareness and participation due to the high levels of skill required. In terms of growth rate, curling and ice hockey had a relatively fast growth rate, followed by skating and luge, while skiing had a relatively low growth rate.

From the perspective of urban agglomeration, in the Beijing–Tianjin–Hebei urban agglomeration, the Baidu Index of curling, ice hockey, and luge had a larger increase, of which, ice hockey's increase in 2022 was as high as 118.92%. In the Yangtze River Delta urban agglomeration, the growth rate of curling also ranked first, with a growth rate of 125.26%. Overall, the growth rate of most winter sports programs in the Yangtze River Delta was higher than that of the Beijing–Tianjin–Hebei, but the Beijing–Tianjin–Hebei growth rate was much higher than that of the Yangtze River Delta in terms of absolute value.

### 4.2 Geographical concentration of the demand for winter sports

The geographical concentration of five winter sports in the Beijing–Tianjin–Hebei and the Yangtze River Delta urban agglomerations is calculated according to formula (1), as presented in Table 3. Notably, there is little difference in the geographical concentration of the five sports. In terms of dynamic trend, the geographical concentration of skating and skiing shows a fluctuating downward trend, which indicates that the two popular winter sports are developing from concentration to decentralization in cities, and the trend is more obvious in the Beijing–Tianjin–Hebei region. However, sports including curling, ice hockey, and luge, which have a slightly higher degree of specialization, show a fluctuating upward trend, and this concentrated trend is more obvious in the Yangtze River Delta. From the perspective of volatility, curling has strong volatility, followed by skating, ice hockey, luge and skiing, which is more obvious in the Beijing–Tianjin–Hebei region.

**Table 3:** Index of centralization in winter in Beijing-Tianjin-Hebei region and Yangtze River Delta region.

year	Beijing-Tianjin-Hebei Region					Yangtze River Delta Region				
	skating	ice hockey	curling	luge	skiing	skating	ice hockey	curling	luge	skiing
2011	0.470	0.450	0.430	0.410	0.540	0.410	0.380	0.400	0.380	0.420
2012	0.450	0.440	0.390	0.400	0.500	0.390	0.400	0.380	0.390	0.410
2013	0.530	0.530	0.480	0.480	0.480	0.470	0.470	0.470	0.450	0.450
2014	0.540	0.560	0.530	0.500	0.470	0.500	0.510	0.530	0.480	0.460
2015	0.560	0.550	0.440	0.500	0.440	0.500	0.500	0.460	0.480	0.440
2016	0.550	0.550	0.440	0.520	0.490	0.500	0.510	0.460	0.490	0.480
2017	0.500	0.520	0.470	0.490	0.480	0.500	0.490	0.490	0.490	0.480
2018	0.490	0.520	0.490	0.490	0.460	0.490	0.510	0.510	0.490	0.440
2019	0.470	0.480	0.450	0.470	0.470	0.500	0.500	0.500	0.490	0.470
2020	0.380	0.410	0.340	0.450	0.410	0.470	0.470	0.480	0.480	0.470
2021	0.410	0.460	0.360	0.470	0.430	0.470	0.480	0.460	0.470	0.470
2022	0.410	0.550	0.640	0.510	0.440	0.400	0.480	0.610	0.510	0.400
mean	0.480	0.502	0.455	0.474	0.468	0.467	0.475	0.479	0.467	0.449
△	−13.64%	20.00%	39.25%	21.74%	−20.41%	−2.47%	23.26%	41.58%	29.21%	−4.88%
S.D	0.06	0.05	0.08	0.04	0.03	0.04	0.04	0.06	0.04	0.03

### 4.3 Specialization of the demand for winter sports

**Fig. 5:**
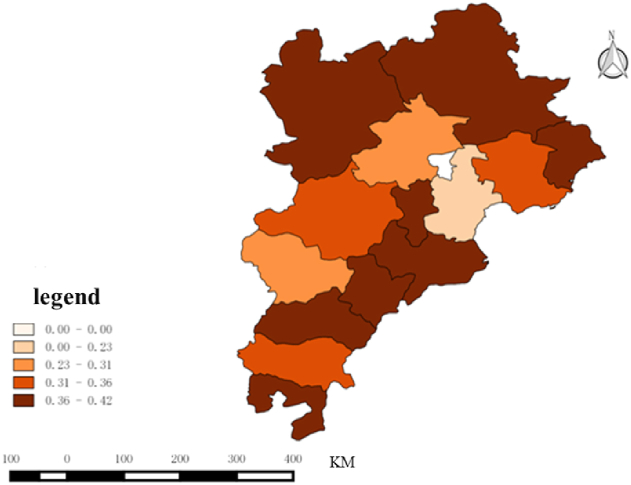
Distribution of specialization index in Beijing-Tianjin-Hebei region from 2011 to 2022.

**Fig. 6:**
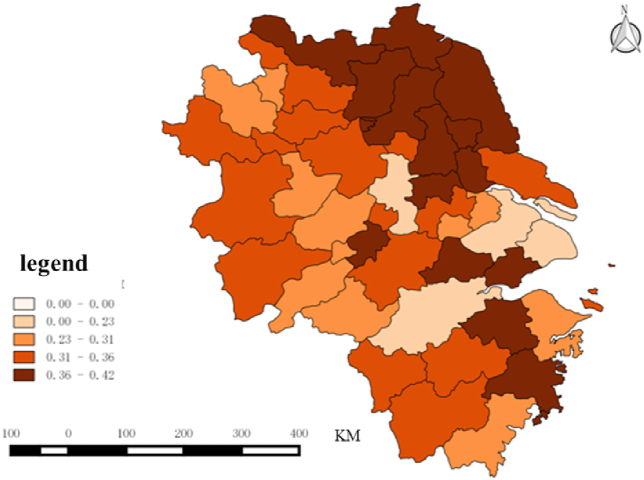
Distribution of specialization index in Yangtze River Delta from 2011 to 2022.

The winter sports specialization index of each city in the two urban agglomerations is calculated according to formula (2), as shown in Table 4, and the spatial distribution is shown in Fig. 5, Fig. 6. Generally, the specialization index of winter sports in the peripheral region of Beijing–Tianjin–Hebei is higher, while the winter sports in Beijing and Tianjin are more diversified. In the Yangtze River Delta urban agglomeration, the specialization index of cities in Jiangsu Province is higher than that of other regions, and Shanghai, Hangzhou, Nanjing, and Suzhou show diversified demand for winter sports.

**Table 4:** Specialization and Trend Classification of the demand for Winter Sports.

	High Growing Trend （≥38%）	Low Growing Trend （<38%）
High Specialization （≥0.336）	Fuyang, Zhoushan, Ma 'anshan, Bengbu, Lishui	Shaoxing, Anyang, Hengshui, Yangzhou, Huzhou, Taizhou, Jinhua, Chengde, Suqian, Nantong, Huai 'an, Jiaxing, Yancheng, Taizhou, Zhenjiang, Wuhu, Changzhou, Baoding, Xingtai, Lianyungang, Cangzhou, Handan, Qinhuangdao, Xuzhou, Langfang, Zhangjiakou
Low Specialization （<0.336）	Shanghai, Huaibei, Chizhou, Hangzhou, Tianjin, Suzhou, Bozhou, Xuancheng, Suzhou, Beijing, Huangshan, Huainan, Anqing, Nanjing, Lu 'an, Quzhou, Ningbo, Tongling, Wenzhou	Shijiazhuang, Chuzhou, Wuxi, Hefei, Tangshan

According to the specialization and growing trend, cities can be divided into four categories: high specialization–high specialization growing trend (H–H), high specialization–low trend (L–L), low specialization–high trend (L–H), and low specialization–low trend (L–L), as depicted in Table 4. It can be seen that most cities are concentrated in H–L and L–H; cities in H–H are mostly peripheral cities of the urban agglomeration such as Fuyang, Zhoushan, and Lishui, and cities in L–L are mainly provincial capitals far from the core cities (Beijing and Shanghai), such as Shijiazhuang and Hefei, and cities that are close to the core cities such as Wuxi and Tangshan.

## 5 Empirical results: did the Beijing Winter Olympics increase the demand for winter sports?

### 5.1 Baseline regression

After dimensionless data, this study adopts the ordinary least squares (OLS) method to estimate the econometric model, and the results are shown in Table 5. Columns (1) to (5) report the results of the influence of interest in the Winter Olympics on the demand for five subitem winter sports, and column (6) presents the Winter Olympics' effect on the total demand for winter sports. It can be seen that the coefficient of the interest in the Winter Olympics in both the demand and total demand for winter sports is all significant at the 1% significance level (*P*-value), which indicates that the increased interest in the Winter Olympics improves residents' demand for winter sports, implying that the holding of the Winter Olympics can drive residents’ participation in winter sports. The goodness of fit (R^2^) of each model is over 0.8, and R squared of the total demand for winter sports is 0.896, indicating that the variables selected in the model of this paper can explain nearly 90% of the changes in the demand for winter sports.

**Table 5:** The results of baseline regression.

	(1)	(2)	(3)	(4)	(5)	(6)
	skating	ice hockey	curling	luge	skiing	total
*OWG*	0.757***	0.67***	0.264***	0.456***	0.429***	0.537***
	(15.07)	(15.53)	(5.84)	(10.02)	(8.43)	(11.48)
*Gdp*	0.356***	0.382***	0.196***	0.384***	0.156***	0.314***
	(8.83)	(11.02)	(5.31)	(10.36)	(3.84)	(8.38)
*Net*	−0.215***	−0.262***	−0.445***	−0.341***	−0.314***	−0.426***
	(-3.31)	(-4.69)	(-7.68)	(-5.85)	(-5.00)	(-7.04)
*Ark*	0.197***	0.153***	0.089***	0.13***		0.117***
	(5.71)	(5.17)	(2.90)	(4.18)		(3.66)
*Tea*	−0.323***	−0.15***	−0.202***	−0.143***	−0.43***	−0.289***
	(-6.91)	(-3.73)	(-3.77)	(-2.66)	(-9.25)	(-6.63)
*Ted*			−0.144**	−0.123**		
			(-2.45)	(-2.08)		
*constant*	0.259***	0.136***	0.382***	0.227***	0.534***	0.36***
*cons*	(6.03)	(3.69)	(5.17)	(3.06)	(15.40)	(9.00)
*N*	486	486	486	486	486	486
*R*^*2*^	0.815	0.896	0.882	0.859	0.837	0.898
*Adjusted R*^*2*^	0.813	0.895	0.881	0.857	0.836	0.896
*F*	358.82	698.90	610.10	422.35	628.79	712.33
*P*	0.0000	0.0000	0.0000	0.0000	0.0000	0.0000

Note: *, ** and *** are significant at 1%, 5% and 10% respectively.

In terms of control variables, the coefficient of economic development, the proportion of mobile internet, and the average temperature are all statistically significant at 1%, the coefficient of air quality is significant in most models except for the demand for skiing, and the coefficient of temperature difference is significant in the demand for curling and luge. The sign of the control variables also concurs with the expectations, in which urban economic development and air quality have a positive influence on the demand for winter sports, while the proportion of mobile internet terminals, average temperature, and temperature difference influence the demand for winter sports negatively. Additionally, interest in the Winter Olympics has a greater influence on winter sports than other factors.

### 5.2 Robustness test

To verify the robustness of the empirical result, after dimensionless the data, this study uses the consistent covariance matrix estimation method (CCME) [49,50] to re-estimate the coefficient. If the significance and goodness of fit of the results are not weakened, and the coefficients of each variable meet the theoretical expectations, indicating that the results in baseline regression are robust. The results of robustness are presented in Table 6. It can be observed that after replacing OLS with CCME, the significance and sign of the coefficient are consistent with the baseline regression, which indicates that the results are still robust after changing the estimation method of the econometric model.

**Table 6:** Robustness.

	(1)	(2)	(3)	(4)	(5)	(6)
	skating	ice hockey	curling	luge	skiing	total
*OWG*	0.757***	0.67***	0.264***	0.456***	0.429**	0.537***
	(8.18)	(10.36)	(3.50)	(4.46)	(3.20)	(5.40)
*Gdp*	0.356***	0.382***	0.196***	0.384***	0.156***	0.314***
	(5.64)	(8.57)	(8.25)	(7.50)	(4.82)	(11.06)
*Net*	−0.215***	−0.262***	−0.445***	−0.341***	−0.314***	−0.426***
	(-4.00)	(-3.44)	(-3.63)	(-3.98)	(-3.46)	(-6.28)
*Ark*	0.197***	0.153***	0.089*	0.13***		0.117**
	(9.04)	(4.30)	(1.53)	(6.09)		(2.55)
*Tea*	−0.323***	−0.15**	−0.202**	−0.143**	−0.43***	−0.289***
	(-6.85)	(-2.64)	(-2.46)	(-2.65)	(-7.27)	(-4.82)
*Ted*			−0.144**	−0.123*		
			(-2.95)	(-1.78)		
*constant*	0.259***	0.136***	0.382***	0.227**	0.534***	0.36***
*cons*	(8.43)	(3.60)	(7.68)	(1.92)	(5.95)	(9.78)
*N*	486	486	486	486	486	486
*R*^*2*^	0.815	0.896	0.882	0.859	0.837	0.898
*Adjusted R*^*2*^	0.813	0.895	0.881	0.857	0.836	0.896
*F*	1832.95	457.51	520.92	11721.80	208.49	733.05
*P*	0.0000	0.0000	0.0000	0.0000	0.0000	0.0000

Note: *, ** and *** are significant at 1%, 5% and 10% respectively.

## 6 Discussion

In this study, applying big data mining techniques, the Baidu Index of Winter Olympics-related terms is used to measure residents' interest in the Beijing Winter Olympics, and the ratio of the Baidu Index of five winter sports to the number of internet searches is used to capture residents' demand for winter sports. Based on an exploration of the spatial–temporal characteristics of the interest in the Winter Olympics and the demand for winter sports, we examined the influence of the hosting of the Beijing Winter Olympics on residents’ demand for winter sports through empirical analyses of data from the two major urban agglomerations of the Beijing–Tianjin–Hebei and the Yangtze River Delta from 2011 to 2019.

Our study expands the research on the socioeconomic benefits of hosting large-scale sporting events. The local influence of sporting events is multifaceted and multilayered, including the enhancement of local visibility, residents' sense of identity and well-being, economic resilience, tourism development, and urban construction [2,3,12]; Gratton et al. et al., 2000 [[6], [7], [8]]; Ulvnes & Solberg, 2016; [10], and there are relatively few studies on the effect of hosting sporting events on residents' participation in sports. The 2022 Beijing Winter Olympics is the most representative large-scale sporting event at the moment, and it is of great practical significance to study its influence on the popularization of winter sports. Our study proves the positive effect of large-scale sporting events on residents’ participation in sports, which affirms the driving effect of hosting the Winter Olympics on winter sports in China.

This study has a few limitations. First, this paper selects two world-class urban agglomerations in China as the research samples, with Shanghai and Beijing as the central cities. Compared with other urban agglomerations in China, they have higher levels of social and economic development and more suitable policies and facilities, which will lead to poor interpretation of the research results for underdeveloped cities. In future research, we will attempt to select a more complete sample of Chinese cities. Second, there may be reverse causality between the main explanatory variables and the explained variables in this study, since a region with a higher rate of sports participation is also more likely to host sporting events, which will inevitably bring about endogeneity issues and pose challenges for identification. In future research, we will focus on finding suitable instrumental variables for hosting sporting events to deal with the endogeneity issues. Third, limited by data availability, this study uses Baidu Index as a proxy variable for winter sports demand, which fails to capture micro-individual information. However, individual heterogeneity is an important factor affecting sports participation, and we will try to incorporate sports big data with micro-individual information to reach more accurate conclusions in later studies.

## 7 Conclusion and implications

In this study, the Baidu Index of Winter Olympics-related terms was used to capture data on residents' interest in the Beijing Winter Olympics and the ratio of the Baidu Index of five winter sports (ice skating, ice hockey, curling, luge, and skiing) to the number of internet searches was used to measure residents’ demand for winter sports. Furthermore, we explored the spatial–temporal pattern of the interest in the Winter Olympics and the demand for winter sports and constructed an econometric model to test the driving effect of the Winter Olympics empirically.

The results are as follows. 1) Since 2011, interest in the Winter Olympics has been on the rise, and the interest shown by residents in Beijing–Tianjin–Hebei has been higher than that of the Yangtze River Delta. 2) The demand for skating and skiing, two popular winter sports, showed a declining geographical concentration, indicating that the popularity of these two sports was on the increase. 3) The demand for winter sports in the peripheral cities in Beijing–Tianjin–Hebei showed a trend of specialization, while Beijing, Tianjin, and some cities in the Yangtze River Delta presented a trend of diversification. 4) The increase in interest in the Beijing Winter Olympics boosted residents’ demand for winter sports significantly, implying that the Winter Olympics successfully drives winter sports participation.

Based on the results, policy implications are proposed.

First, with the help of sporting events, residents' participation in sports should be promoted. According to the results of temporal and spatial analysis, it can be seen that the hosting of the Beijing Winter Olympics, as well as related national policies and industries, all played a certain incentive role for Chinese residents to participate in winter sports. The Beijing–Tianjin–Hebei region had a higher interest in the Winter Olympics due to the combination of the proximity of the organizing site and the climate conditions. However, Shanghai, Hangzhou, Nanjing, Suzhou, and other cities also showed a demand for ice and snow sports. Therefore, for the cities in the core area of the Yangtze River Delta, the construction of ice and snow venues can be accelerated, various types of ice and snow events can be held, and independent ice and snow event brands can be cultivated to promote the development of the ice and snow industry and improve the level of residents’ participation in sports to realize the goal of universal participation in ice and snow sports.

Second, a specialized sports industry should be built according to local conditions. From the empirical results, the factors that affect residents’ participation in winter sports are not only large-scale events but also environmental factors such as air quality and temperature. North of the Yangtze River, owing to the natural advantages of temperature, snow and ice sports development conditions are more favorable, while the Yangtze River Delta and other urban agglomerations located in the southern region do not have the natural endowment advantages of snow and ice sports, but these regions do have the advantages of a developed economy and a high degree of openness to outside world. Each region should develop special sports programs according to environmental and cultural characteristics to avoid the formation of homogeneous and inefficient competition.

Third, publicity should be strengthened to stimulate the enthusiasm of residents for sports. Based on the results of the study, when the proportion of internet mobile terminals in a region increases, the demand by residents for winter sports will decrease, which indicates that short videos, hand games, and other forms of leisure and entertainment that need to rely on the mobile internet and sports show a competitive relationship between substitution and publicity. Not only snow and ice sports but also other sports need residents to have enthusiasm for sports and thereby enhance their participation; therefore, the state and the region should unite with the local sports departments to engage in a variety of forms of publicity, including sporting events, combined with the current fashion elements, to popularize and promote all kinds of sports and sports programs successfully, to help familiarize more people with various sports, to stimulate the enthusiasm of residents for sports and to enhance their participation.

## Data availability statement

Data will be made available on request.

## Additional information

No additional information is available for this paper.

## CRediT authorship contribution statement

**Peipei Wu:** Conceptualization, Formal analysis, Writing – original draft. **Xiaochuan Zhu:** Data curation, Methodology, Supervision, Writing – original draft. **Shuqian Yang:** Data curation, Software, Validation, Visualization. **Junpei Huang:** Formal analysis, Methodology, Visualization, Writing – review & editing.

## Declaration of competing interest

The authors declare that they have no known competing financial interests or personal relationships that could have appeared to influence the work reported in this paper.
